# The relationship between prolonged travel and mortality among women with complicated deliveries in rural Kenya

**DOI:** 10.7189/jogh.16.04300

**Published:** 2026-09-18

**Authors:** Nicholas Errol Rahim, Margaret E Kruk, Jan E Cooper, David Kapaon, Kennedy Opondo, Kevin Croke

**Affiliations:** 1 Department of Global Health and Population, Harvard T.H. Chan School of Public Health, Boston, Massachusetts, USA; 2 Department of Medicine, Division of General Medicine and Geriatrics, Washington University School of Medicine, St. Louis, Missouri, USA; 3 Department of Epidemiology, Biostatistics and Occupational Health, McGill University, Montreal, Quebec, Canada; 4 Department of Epidemiology, Boston University School of Public Health, Boston, Massachusetts, USA; 5 Department of Global Health and Population, Harvard T.H. Chan School of Public Health, Kisumu, Kenya

**Keywords:** intrapartum, travel distance, maternal and child health, delivery care, delays in care

## Abstract

**Background:**

In many low- and middle-income settings, deliveries often take place in primary health facilities that are not equipped to address life-threatening complications. Emergency referral systems seek to rapidly transport women and newborns facing complications but inevitably introduce delays. Estimating the mortality impact of these delays is challenging due to potential selection bias. As an alternative approach, we examine the relationship between delays and adverse outcomes among a sample that is less likely to exhibit selection effects: mothers who started at a low-level facility before transfer to a hospital equipped to provide definitive care (hospital-level).

**Methods:**

We leverage demographic, obstetric, and geodata collected through the parent study Service Delivery Reform for Maternal and Newborn Health in Kakamega County, Kenya to explore the relationship between intrapartum travel and whether there was a maternal death, neonatal death, or stillbirth (adverse health outcome). We conducted a series of logistic models estimating the relationship between longer travel distances and probability of an adverse health outcome, with analyses iteratively adjusted for sociodemographic and obstetric covariates.

**Results:**

Of the 39,052 mothers with full geodata, 1,928 (4.9%) reported visiting at least one low-level facility before visiting a high-level facility. After restricting to those who experienced upward movement in facility level during delivery and adjusting for relevant covariates, we find that those in the fourth quartile of travel distances (42–436 km) had a 2.60 times higher odds of an adverse health outcome relative to those in the first quartile (2–16 km).

**Conclusions:**

Our findings show that Phase 2 delays in the form of intrapartum journeys have a pronounced association with maternal and child mortality when the total distance travelled is long. These findings align with the prior evidence that delays in receiving appropriate and timely delivery care are related to maternal and child mortality and morbidity.

Despite global declines in maternal and newborn mortality since the turn of the 21st century, the pace of progress has slowed in recent years [1,2]. Moreover, the burden of maternal mortality is not equitably distributed across the world. Sub-Saharan Africa accounts for 70% of maternal deaths globally [3]. The region also faces the highest neonatal mortality rate (NMR), with 27 neonatal deaths per 1000 livebirths [4]. Deliveries in many low- and middle-income settings often take place in primary health facilities that are not fully equipped to address life-threatening complications, as demonstrated by the inverse relationship between the share of facility-based deliveries that occur in hospitals and neonatal mortality [5]. Emergency referral systems seek to rapidly transport women and newborns facing such complications to facilities that can provide life-saving care. But in many cases, referral systems do not reliably provide rapid transport [6]. Previous studies across national contexts have shown that delays during the delivery episode are major contributors to amenable mortality for both mothers and newborns [7–10].

In 1994, Thaddeus and Maine proposed the ‘Three Delays’ model in order to conceptualise the phases in which a delay can influence health outcomes:

• Phase 1: a delay in the decision to seek care;

• Phase 2: delay between the decision to seek care and arrival to a health facility capable of providing appropriate care; and

• Phase 3: delay between arrival at the health facility and receipt of care [11].

This paper focuses on Phase 2 delays as quantified by the total distance travelled during the delivery episode. Phase 2 delays do not pertain simply to the arrival at any health facility, but rather at a health facility that is equipped with resources needed to address both expected and unexpected delivery complications. A minimal standard would include trained physicians, capacity to perform caesarean sections, capacity to perform blood transfusions, and relevant medications such as misoprostol and magnesium sulphate [12–15].

Kenya is an important setting in which to consider this topic. While Kenya has made major progress over the past twenty years in the reduction of under-five mortality, maternal and newborn mortality have improved more slowly. Over the past decade, Kenya has also undergone a major health system decentralisation, in which county governments control primary care services. Health facilities in Kenya are categorised into six levels, with the first three levels comprising primary healthcare while levels 4–6 denote hospital-based specialised care [16]. Due to Kenya’s decentralisation reforms, county governments are now responsible for referral from first line facilities to hospitals. Current national guidelines on when a patient should be referred to a higher level facility during a delivery episode are not fully defined, with referral recommended when a patient experiences ‘complications’ in a low-level facility [17]. Kakamega County implemented a new county-level referral policy on 31 January 2025, which calls for improvements to the infrastructure, ambulatory equipment, and lines of communication necessary for efficient referral of patients [18]. However, while this policy provides general guidelines for referral, it is not specific to labour and delivery. While there is no clear national or county-level definition of what constitutes an intrapartum complication requiring referral, the World Health Organization (WHO) details potential symptoms of complications that would require emergency obstetric services, which include shock, foetal distress, heavy bleeding, and malpresentations of the foetus [19]. A lack of a clear protocol for referral during delivery at the national level may pose particular challenges in contexts like Kenya, where 35% of deliveries occur in low-level facilities or at home and providers may face difficult decisions about the appropriateness of referral from these settings [20]. Another cause for intrapartum movement between facilities is provider non-responsiveness, such as when labouring individuals are turned away after presenting to an initial facility. Non-responsiveness in this setting can be due to lack of capacity to accommodate an additional patient due to infrastructure or personnel constraints, ongoing health worker strikes, and/or provider mistreatment [21].

Studying the impact of referral on health outcomes is challenging, since intrapartum movement between facilities is typically itself an indicator of medical complications. As such, referred women tend to be at higher risk than women who are not referred. A naive analysis of the relationship between referral and maternal and child health outcomes would be susceptible to confounding bias from unobservable underlying health risks. While prior research has explored the associations between each of the three categories of delay and adverse outcomes, many studies fully or partially limit their sample to women who experience severe maternal morbidity or mortality. Yet these outcomes are themselves potential downstream consequences of delays; therefore restricting analysis to this sample can introduce selection bias into results [22].

In order to approach this issue from another angle, we conduct analysis on a sample which is less likely to exhibit selection effects, while also seeking to minimise other potential confounders. Our primary sample is restricted to delivering mothers who moved from at least one low-level facility to a high-level facility (henceforth referred to as ‘upward movement in facility level’ (UMFL)), without any sample restriction based on their ultimate case outcome. Restricting the analysis to women who experienced UMFL reduces confounding by indication arising from comparisons between women who required higher-level care and those who did not. It therefore allows us to examine variation in outcomes among women who experienced broadly comparable escalations in level of care. In sensitivity analyses, we further restrict the sample to respondents who end their delivery at the same referral facility (the County’s highest-level referral hospital), to further reduce potential confounding by indication.

By constructing our primary sample based on a major contributor to total Phase 2 delays (UMFL), we can better explore the relationship between intrapartum travel pathways and adverse health outcomes. This in turn may help policymakers understand whether improvements to referral practices can be an effective tool in improving maternal and child health (MCH). In this paper, we investigate the association between mortality, stillbirth, or neonatal mortality and intrapartum travel distance among those mothers who visited at least one low-level facility in labour prior to delivering at a referral facility.

## METHODS

### Study setting

The data analysed in this study were collected as part of an evaluation of the Service Delivery Redesign for Maternal and Newborn Health (SDR) programme implemented in Kakamega County, located in western Kenya [23]. Kakamega has an estimated population of 1.9 million and an area of 3033 km^2^. Approximately 70,000 births took place in the county in 2018 [24]. As a part of the SDR evaluation, 72 healthcare facilities that provide antenatal care (ANC) were selected across all 12 of Kakamega’s sub-counties as sites for participant recruitment. The probability of a facility being selected for inclusion was proportional to the volume of ANC visits, with sampling stratified at the sub-county level. A facility was ineligible for selection if it was located in Kakamega town, privately operated, or delivered ANC services to less than six new clients each month. We refer to facilities level 1–3 as low-level and facilities level 4–6 as high-level.

The reporting of this manuscript follows the STROBE guidelines (Appendix 23 in the **Online Supplementary Document**).

### Study population

Participants were enrolled between February 2022 and April 2025 across the 72 included facilities. The data collection team (Ipsos-Kenya) sought to screen every woman presenting for ANC in person at the selected facilities for the parent study. Participants were excluded from participating if they reported intentions to deliver outside of Kakamega county, at a referral facility due to pre-existing conditions, or were less than 15 years of age.

For this secondary analysis, the sample was limited to those with complete geospatial data for home village and facilities visited during delivery and those who reported living in Kakamega County. Because we must assume that every participant’s delivery episode begins in the home village, we exclude those participants with a mapped travel distance between home village and the first facility visited that is equal to or greater than 111 km, which is the longest straight-line path between two points within the county.

### Data collection

During in-person enrolment, research staff collected information on participants’ basic demographics, obstetric history, and antenatal care and diagnoses. Participants were recontacted by phone starting from seven and 28 days post expected delivery date to collect data on delivery episode, maternal and child health outcomes, and postnatal care receipt. If participants were unable to be reached by phone, follow-up interviews were conducted in person.

### Variables of interest

The primary outcome of interest is a binary variable that indicates whether the mother or newborn experienced either maternal mortality, neonatal mortality, and/or stillbirth. The primary explanatory variable is travel distance measured in km. Distances were calculated using longitude and latitude coordinates of health facilities in Kakamega and respondents’ home villages and the R package ‘OSRM’ to interface with OpenStreetMap-Based Routing Service, which returns the distance of the optimal travel route between two points given observed road networks [25]. More details on the geocoding process, along with other data cleaning choices, is provided in Appendix 1 in the **Online Supplementary Document**. These calculations account for travel from the participant’s home village to the first health facility, and from the first facility to the second facility, and so on. Total travel distance was then calculated by summing all travel distances for each portion of the journey to the final facility. In this calculation, we assume visiting a low-level facility after visiting a high-level facility reflects immediate post-partum care visits. As such, we cut off intrapartum travel routes at the final high-level facility reported for those who report visiting at least one high-level facility during their delivery episode. We relax this assumption in a sensitivity analysis. For those participants who only report only visiting low-level facilities, all individual travel paths between facilities are included in estimating the total distance travelled.

Relevant covariates that predict maternal and early child mortality include maternal age, maternal educational attainment, distance between home village and nearest high-level facility, number of ANC visits, whether the delivery concluded a multiple gestation, prior history of caesarean section, prior history of miscarriage, and a composite variable for gestational complications indicating whether the mother reported anaemia, diabetes, or hypertension during pregnancy. In the absence of comprehensive medical records available to the research team, we assume that reported UMFL serves as a proxy for complications requiring emergency referral to a higher-level facility. While this measure is imperfect, there is evidence in our data that UMFL is an appropriate proxy. Among those who experienced UMFL, 37.5% reported at least one delivery procedure that would require referral from a low-level facility (caesarean section, hysterectomy, intubation, or blood transfusion), compared to the 9.1% prevalence among the non-UMFL group.

### Analysis

**Figure 1:**
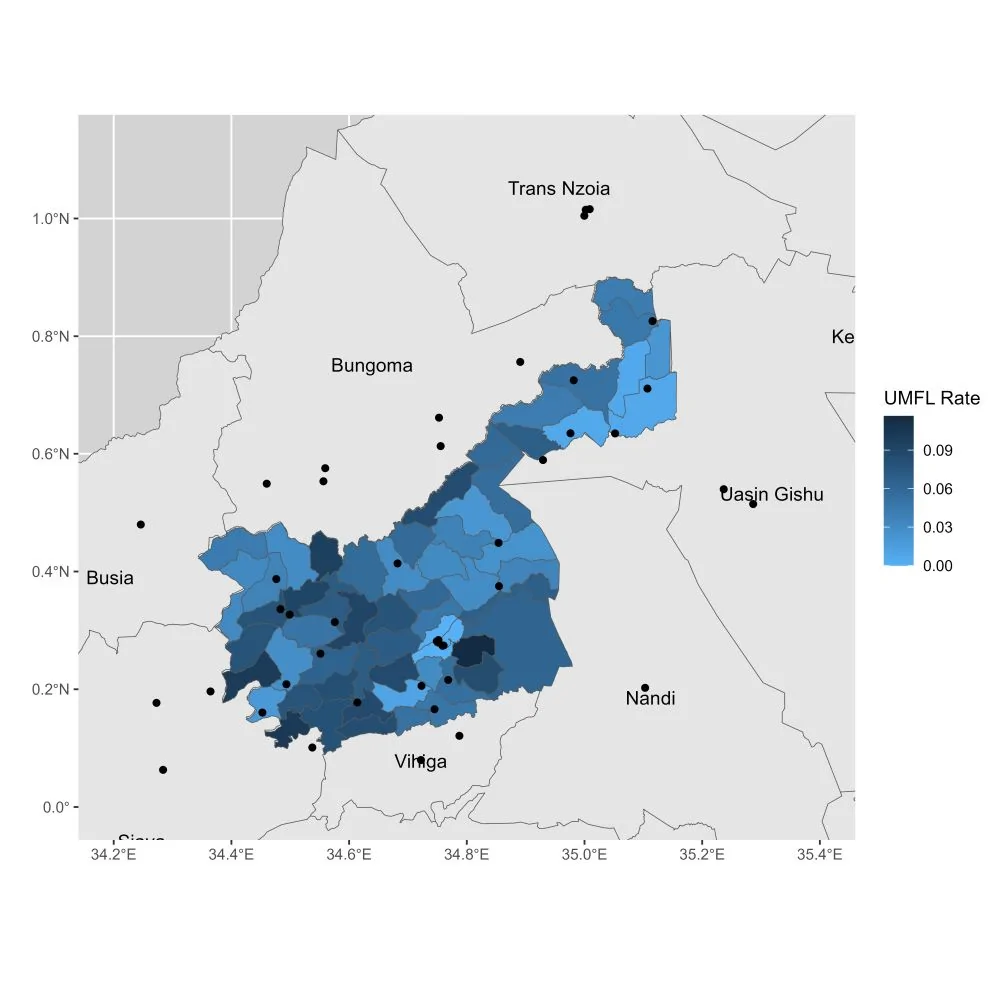
Prevalence of UMFL during delivery episode in Kakamega County, aggregated at the ward-level. Colour represents the prevalence of referral from low-level facility to high-level facility by ward, with darker shades indicating higher prevalences. Black dots indicate the locations of high-level facilities that appear at least three times in the data. UMFL – upward movement in facility level.

We summarise sociodemographic and obstetric characteristics among those who experienced UMFL in **Table 1**. We also present distance between the participant’s home village to the nearest high-level facility and the total intrapartum travel distance. Differences in characteristics by quartiles are assessed by ordinal logistic regression models for categorical and dichotomous variables or linear regression models for continuous variables. Models with binary indicators for quartile were tested for statistical differences from null models by likelihood-ratio tests and F-tests. To illustrate the geographic distribution of UMFL, we present a map indicating the rate of UMFL by ward, which is the most granular administrative unit within counties in Kenya (**Figure 1**). We estimate the relationship between UMFL and relevant covariates *via* both bivariate and fully adjusted logistic regressions.

**Table 1:** Demographic and obstetrics characteristics by intrapartum travel distance quartiles among UMFL group*

Characteristic	1st quartile (n = 484)	2nd quartile (n = 480)	3rd quartile (n = 486)	4th quartile (n = 478)	*P*-value
Age					
*19 years or younger*	20% (98/484)	20% (94/480)	20% (99/486)	23% (108/478)	0.649
*20–34 years*	70% (341/484)	71% (341/480)	70% (341/486)	69% (330/478)	
*35 years or older*	9% (45/484)	9% (45/480)	9% (46/486)	8% (40/478)	
Education					
*Did not complete primary*	18% (87/479)	13% (61/475)	14% (67/481)	15% (72/475)	0.006
*Completed at least primary*	46% (221/479)	46% (218/475)	42% (204/481)	49% (231/475)	
*Completed at least secondary*	36% (171/479)	41% (196/475)	44% (210/481)	36% (172/475)	
Distance to nearest high-level facility (km)	7.01 (3.17)	9.51 (3.66)	8.02 (3.97)	7.87 (5.2)	<0.001
Antenatal care					
*<4 visits*	17% (83/484)	17% (83/480)	15% (73/486)	19% (91/477)	0.658
*4–7 visits*	75% (364/484)	77% (369/480)	78% (378/486)	73% (350/477)	
*8 or more visits*	8% (37/484)	6% (28/480)	7% (35/486)	8% (36/477)	
Multiple gestation	5% (26/484)	4% (19/480)	5% (23/486)	6% (27/478)	0.472
Previous caesarean	6% (29/484)	14% (66/480)	12% (57/486)	11% (53/478)	0.009
Previous miscarriage	17% (80/484)	18% (84/479)	18% (86/486)	17% (81/478)	0.949
Gestational complications	6% (31/484)	9% (43/480)	7% (35/486)	9% (43/478)	0.306
Intrapartum travel, mean (km)	11.38 (3.34)	20.64 (2.38)	33.10 (4.95)	70.51 (33.46)	-
Intrapartum travel, range (km)	2.4–16.4	16.4–25.3	25.3–42.3	42.3–435.7	-

UMFL – upward movement in facility level

*Results reported as percentage who reported affirmative response (number of affirmative responses/total number of non-missing responses) or mean (standard deviation). *P*-values are calculated from ordinal logistic regression models or linear regression models accounting for standard errors at the enrolment facility-level, and conducting likelihood-ratio tests/F-tests comparing to null models.

Finally, we restricted the analysis to only respondents who experienced UMFL and estimated a series of logistic models estimating the relationship between travel distance and probability of an adverse health outcome, with analyses iteratively adjusted for sociodemographic and obstetric covariates. We also re-estimated this set of logistic regressions in a set of sensitivity analyses with the following variations in the analytical sample:

1) respondents who reported multiple facilities during intrapartum;

2) respondents who reported multiple facilities during intrapartum, removing those who reported visiting low-level facilities after high-level facilities;

3) respondents who experienced UMFL, but no longer restricting to only those who reported living in Kakamega County or whose first leg of their journey was equal to or less than 111 km; 

4) only respondents who visited Kakamega County General Hospital as their final facility;

5) only respondents whose final delivery facility was level 5 or 6;

6) excluding respondents those who reported living in sub-counties that were designated to receive the Service Delivery Redesign health system intervention.

We also conducted a sensitivity analysis in which we removed the largest reported travel distance in order to avoid outlier-driven results. All regressions were estimated with robust standard errors that account for clustering at the ANC facility-level. We analysed using Stata, version 18.0 (StataCorp, College Station, TX, USA).

## RESULTS

**Figure 2:**
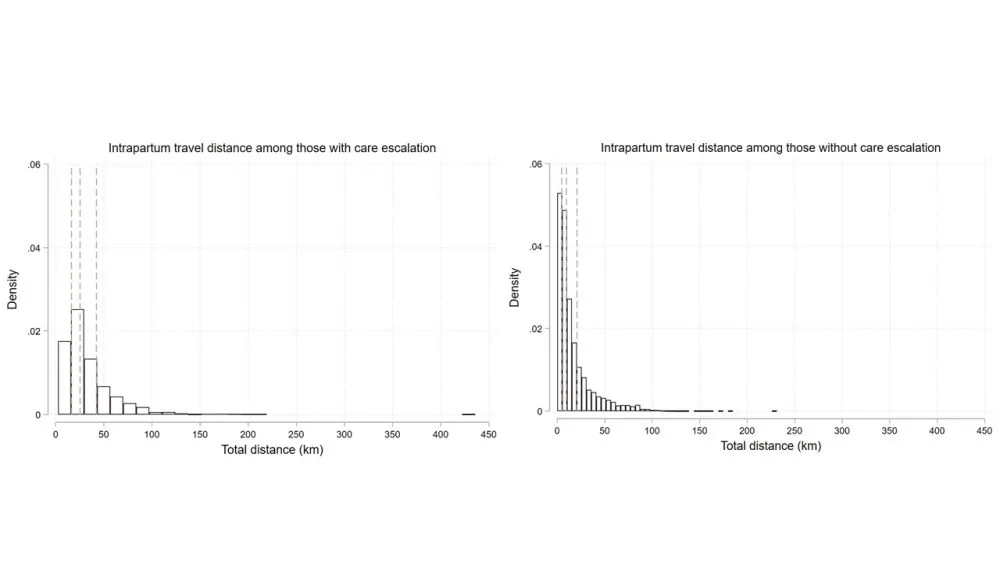
Distributions of total intrapartum travel distances by UMFL group. Panel A. Intrapartum travel dis­tance among those with care escalation. Panel B. Intrapartum travel distance among those without care escalation. Pink lines represent thresholds of quartiles. UMFL group: quartile 1 = 2.4–16.4 km; quartile 2 = 16.4–25.3 km; quartile 3 = 25.3–42.3 km; quartile 4 = 42.3–435.7 km. No UMFL group: quartile 1 = <0.1–4.7 km; quartile 2 = 4.7–9.7 km; quartile 3 = 9.7–20.8 km; quartile 4 = 20.8–231.1 km. UMFL – upward movement in facility level.

A total of 60,944 participants were enrolled into the parent study. Of these 60,944 participants, 40,304 (66.1%) had full facility and home village geocode data. Of those with full geocode data, 39,052 (96.9%) live in Kakamega County and reported a first leg of their intrapartum journey less than or equal to 111 km. This leaves a sample in which 1,928 (4.9%) respondents reported UMFL during their delivery episode, while 37,124 (95.1%) did not. A total of 751 (1.9%) participants experienced an adverse health outcome. The most common final facility visited in the UMFL group was Kakamega County General Hospital (25.5%). Among those who moved to a higher-level facility, 94.0% visited two facilities, 5.5% visited three facilities and less than 0.5% visited four or more facilities. Estimated travel distances for the UMFL group ranged between 2.4 and 16.4 km for the first quartile, 16.4 and 25.3 km for the second quartile, 25.3 and 42.3 km for the third quartile, and 42.3 and 435.7 km for the fourth quartile. Distributions of travel distances for those who did and did not experience UMFL are presented in **Figure 2**.

With an alpha-threshold of 0.05, there was a significant difference between quartile groups by educational attainment. Additionally, those in the second quartile group lived farther from a high-level facility (quartile 1 = 7.01 km; quartile 2 = 9.51 km; quartile 3 = 8.02 km; quartile 4 = 7.87 km) and were more likely to have a prior history of caesarean section (quartile 1 = 6%; quartile 2 = 14%; quartile 3 = 12%; quartile 4 = 11%) (**Table 1**). UMFL rates by ward ranged from 0% (Mahiakalo, Sheywe, and Shirere in Lurambi sub-county) to 11.7% (Murhanda in Shinyalu sub-county) (**Figure 1**).

In the fully adjusted logistic regression modelling the factors associated with UMFL, age 20–34 years was associated with an odds ratio of 0.80 (95% confidence interval (CI) = 0.70–0.91), relative to those aged 19 years or younger. Additionally, multiple gestation pregnancy was associated with an odds ratio of 3.24 (95% CI = 2.49–4.22), history of prior caesarean section was associated with an odds ratio of 2.61 (95% CI = 2.02–3.39), and history of previous miscarriage was associated with an odds ratio 1.14 (95% CI = 1.00–1.29) (**Table 2**).

**Table 2:** Univariable and fully adjusted logistic regression models estimating the relationship between the likelihood of UMFL during delivery episode and selected risk factors*

	Unadjusted		Adjusted	
**Characteristic**	**Odds ratio (95% CI)**	***P*-value**	**Odds ratio (95% CI)**	***P*-value**
Age				
*20–34 years*	0.88 (0.78–0.98)	0.027	0.80 (0.70–0.91)	0.001
*35 years or older*	0.91 (0.71–1.17)	0.461	0.80 (0.61–1.04)	0.094
Education				
*Completed at least primary*	1.11 (0.97–1.28)	0.133	1.11 (0.95–1.29)	0.201
*Completed at least secondary*	1.09 (0.90–1.33)	0.385	1.12 (0.91–1.38)	0.281
Distance to nearest high-level facility (km)	1.02 (1.00–1.05)	0.050	1.02 (1.00–1.05)	0.055
Antenatal care	1.00 (0.88–1.13)	0.983		
*4–7 visits*	1.06 (0.82–1.37)	0.653	0.99 (0.86–1.13)	0.838
*8 or more visits*			1.03 (0.79–1.35)	0.832
Multiple gestation	3.31 (2.58–4.24)	<0.001	3.24 (2.49–4.22)	<0.001
Previous caesarean	2.51 (1.94–3.26)	<0.001	2.61 (2.02–3.39)	<0.001
Previous miscarriage	1.13 (0.99–1.28)	0.066	1.14 (1.00–1.29)	0.048
Gestational complications	1.06 (0.88–1.28)	0.560	1.05 (0.87–1.26)	0.613

CI – confidence interval, OR – odds ratio, UMFL – upward movement in facility level

*All models estimated with standard errors adjusted for clustering at the enrolment antenatal facility. Gestational complications include diagnosis of anaemia, hypertension, and/or diabetes during pregnancy.

**Figure 3:**
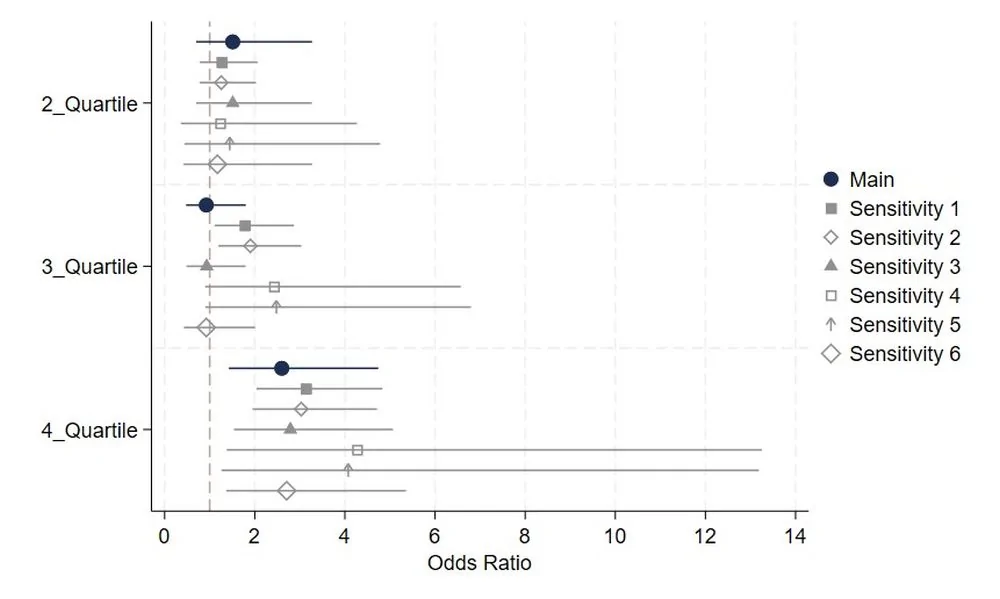
Logistic regressions estimating relationship between total travel distance during delivery episode categorised in quartiles and the likelihood of an adverse health outcome. All models adjusted for demographic and obstetric characteristics and estimated with standard errors accounting for clustering at the enrolment antenatal facility. Bars indicate 95% confidence intervals for estimated odds ratios. Reference category: 1st quartile. Sensitivity model 1: sample was restricted to those who reported multiple facilities during intrapartum. Sensitivity model 2: sample was restricted to those who reported multiple facilities during intrapartum and the assumption that low-level facilities reported after high-level facilities were reporting errors was relaxed. Sensitivity model 3: sample was no longer restricted to only those who reported living in Kakamega County and whose first leg of their delivery journey was equal to or less than 111 km. Sensitivity model 4: sample was restricted to those who visited Kakamega County General Hospital as their final facility. Sensitivity model 5: sample was restricted to those whose final facility reported was level 5 or 6. Sensitivity model 6: sample excludes those who reported living in those sub-counties that were designated to receive the health system intervention as a part of Service Delivery Redesign for Maternal and Newborn Health. UMFL – upward movement in facility level.

Finally, in the analyses restricted to the UMFL group, fourth quartile total travel distance is associated with higher odds of experiencing an adverse health outcome relative to those with travel distances in the first quartile, ranging from 2.49 (95% CI = 1.42–4.37) in the semi-adjusted model to 2.60 (95% CI = 1.43–4.74) in the fully adjusted model (Appendix 7 in the **Online Supplementary Document**). These findings remain qualitatively unchanged in sensitivity analyses (Appendix 8–13 in the **Online Supplementary Document**). The estimated odds ratios for each quartile from the fully adjusted main analysis and sensitivity analyses are illustrated in **Figure 3**.

## DISCUSSION

Our primary analysis shows that even among those women with complex intrapartum travel pathways, those who travelled farther faced significantly higher odds of experiencing maternal mortality, neonatal mortality, and/or stillbirth. It remains challenging to estimate the causal impact of referral, as a patient’s health condition is typically correlated with both upward movement in facility level and final facility destination. We limit this confounding by restricting our sample to respondents who follow similar intrapartum pathways. Furthermore, the durability of the estimates across sensitivity analyses, including those which hold the final facility constant, improves our confidence that this association we are observing is not solely driven by confounding between the total travel distance and unobserved individual risk factors. This positive association between travel distance and adverse health outcomes is in line with prior literature that demonstrates an association between delays during childbirth and worse health outcomes for mother and child.

Our results deviate from some of the prior literature in that we find travel distances, which we categorise as a Phase 2 delay, to be associated with worse health outcomes. This is inconsistent with Benimana *et al*. (2018) who find that, among cases of maternal near-misses and mortality from a teaching hospital in Rwanda, few were attributable delays in reaching higher level care. Rather, the researchers attribute the majority to Phase 3 delays [7]. Similarly, in a sample of near-miss cases in a regional hospital in Afghanistan, Hirose *et al*. (2012) find no association between foetal death and Phase 2 delays. Instead, they find significant differences in regard to Phase 1 delays [9]. One possible explanation for the discrepancy between these findings and ours is that the two highlighted studies identified observations based on whether they presented at a specific high-level facility and experienced life-threatening complications during delivery. It is possible that conditioning inclusion into the analytical sample on near-miss status, as these studies have done, blocks a key causal pathway between intrapartum care delays and other adverse health outcomes, particularly newborn mortality. This would lead to the estimate of the relationship between health outcomes and Phase 2 delays to be biased toward the null. Additionally, there is some evidence that Phase 2 delays due to transfers between health facilities can result in Phase 3 delays. In health systems across low income settings, previous literature has highlighted that a lack of clear and enforced referral guidelines can result in women being referred for life-threatening complications, but not being attended to promptly at the receiving facility [6].

This analysis also shows that multiple gestation pregnancy has the highest association with both UMFL and adverse health outcomes. Multiple gestation is a known risk factor for maternal and child mortality and morbidity [26–28]. In this sample, multiple gestation is 4.9% in the UMFL group *vs.* 1.5% in the comparison group, indicating that many women are not initiating labour at appropriate facilities for complex deliveries. Improved diagnostic services and targeted counselling of mothers carrying twins during antenatal care could reduce the need for high-risk transfers for these mothers.

A strength of this study is that we leverage data from a large sample of women who were screened for inclusion to the parent study during pregnancy, who were then followed up during early postpartum. This allows for a more precise and granular recollection of the trips taken during intrapartum. Through our geocoding, we are able to more accurately map out these trips to estimate the distance travelled. However, there are also limitations to this analysis. While we have detailed data on Phase 2 delays in terms of travel distances, we have more limited data on Phases 1 and 3 delays. Additionally, while the latent variable we are interested in is the length of time between deciding to seek care and arriving at a facility equipped to deliver appropriate care, our independent variable measures physical distance based on optimal routing. Despite the likelihood of a strong correlation between travel distance and time elapsed, the former cannot perfectly capture the latter. Moreover, we can only estimate the optimal route between points based on existing road networks, rather than the actual distance travelled. It is possible that discrepancy between our estimation and actual travel distance increases the farther away points are from each other geographically, as the number of potential routes increases and the individual’s familiarity with routes decreases with distance from their home village. However, bias from this potentially differential measurement error is minimised by categorising total travel distance into quartiles.

While we can observe whether an individual moved from a low-level facility to a high-level facility, we cannot determine the reason for this movement. Such movement can result from official referrals due to complications, being turned away from the low-level facility due to unavailable services, or if the individual chooses to transfer to another facility because of poor service. Each scenario would have different implications for the degree to which we are underestimating the totality of Phase 2 delays. While we assume that UMFL is correlated with clinical referrals during delivery, we cannot say so definitively. As such, it is possible that despite restricting the analysis to those who reported UMFL and adjusting for relevant observables, there are unmeasured risk factors that are correlated with distance travelled and likelihood of adverse health outcomes that can bias results. If we assume that these risk factors are positively correlated with both travel distance and health outcomes, then our results would be biased away from the null hypothesis. Another limitation of this study is a lack of data on the ordering of adverse events and travel. Despite having information on whether the individual experienced any mortality event and on all facilities visited during child delivery, we do not know if events occurred before or after the total travel journey was completed. As such, it may be the case that the occurrence of a mortality event in the first facility prompted referral to another, farther away facility. Further research that can better document the magnitude of each delay type, reason for movement between facilities, and the temporal ordering of events in relation to travel would provide more insight into this issue.

Finally, Kakamega County may not be representative of the rest of Kenya. The county has experienced numerous changes in the past few years, including the Service Delivery Redesign reform and the increased participation of private ambulance services [29–31]. As such, results from this study may not generalise to other settings.

## CONCLUSIONS

Our findings show that Phase 2 delays in the form of intrapartum journeys have a pronounced association with maternal and child mortality when the total distance travelled is long, >40 km (Appendix 5 in the **Online Supplementary Document**). If we focus on Phase 2 delays from prolonged travel due to inter-facility movement, there are two possible reforms that could reduce the total distance needed to travel for women who experience unexpected and severe complications during delivery. First, this issue can be mitigated if there is a detailed county-level policy that explicitly states the criteria for intrapartum referral and requires low-level facilities to have pre-identified referral pathways that strike the optimal balance between the quality of the receiving facility and minimal distance needed to travel. To take this further, local health systems can establish networks of low-level, satellite facilities centred near well-equipped, high-level facilities and encourage patients to begin their delivery care at these satellite facilities [32]. This would further minimise the potential distance needed to travel during the delivery episode in the event of complications requiring referral. The second, more radical solution would be a reform in line with SDR that moves more deliveries to begin care at higher-level facilities that have been improved such that they can handle this increase in volume. In this scenario, the need for referral itself would be reduced and, in turn, reduce the total distance needed to travel. However, as prior literature has expressed, this would require significant, sustained administrative and financial investment from local government in order to be effective [33,34].

## Additional material


Online Supplementary Document

## Data Availability

**Data availability:** Data will be made available after the SDR evaluation study is completed.
