# Supplementary material for: The relationship between prolonged travel and mortality among women with complicated deliveries in rural Kenya
Source: J Glob Health. 2026 Sep 18;16:04300. doi: 10.7189/jogh.16.04300 (PMC13587161; doi:10.7189/jogh.16.04300)
Supplement: Supplementary file 1 [file jogh-16-04300-s001.pdf]

Supplement to: Rahim NE, Kurk ME, Cooper JE, Kapaon D, Opondo K, Croke K. The relationship between prolonged travel and mortality among women with complicated deliveries in rural Kenya. J Glob Health. 2026;16:04300.

Appendix 1. Process for producing geocodes for villages and health facilities and home subcounty cleaning

During study procedures, enumerators ask participants about their home village, ward, and subcounty, in addition to which health facilities they visited during the intrapartum period. Using this data, villages and health facilities are matched with official lists of villages and health facilities from the Kakamega County government that contain the geocodes (latitude and longitude) of these locations. A large number of village locations reported by respondents were not present in the official list, often because respondents provided the name of a local landmark (e.g. primary school, or church) to mark their location, rather than the official village name. For those village names/locations that were reported by at least five participants, but which were not included in the official list, on-site study personnel confirmed their locations and manually produced geocodes for these villages using the online mapping service Google Maps. Geocodes for villages within Kakamega were further reviewed if they placed the village more than 10 km outside of Kakamega County. Those villages with missing geocodes were left as missing without imputation. The coordinates of health facilities were received from Kakamega County health authorities. Health facilities without geocodes listed by the county were manually geocoded if they appeared at least 3 times in the data as a facility visited during child delivery. These geocodes were obtained from the Kenta Master Health Facility Registry and, if not available there, from Google Maps. For those who reported a home ward that was not located in the reported subcounty, the subcounty was replaced with the subcounty that corresponded with the ward reported. This choice was made under the assumption that participants are more likely to know their ward rather than subcounty. This alteration applies to 0.1% (80/60,944) of observations.

Appendix 2. Demographic and obstetrics characteristics by UMFL group.

Characteristic	No UMFL (N = 37,124)	UMFL (N = 1,928)	p-value
Age			
19 years or younger	19% (6,927/37,124)	21% (399/1,928)	0.194
20 - 34 years	72% (26,843/37,124)	70% (1,353/1,928)	
Older than 34 years	9% (3,354/37,124)	9% (176/1,928)	
Education			
Did not complete primary	16% (5,932/36,315)	15% (287/1,910)	0.611
Completed at least primary	45% (16,207/36,315)	46% (874/1,910)	
Completed at least secondary	39% (14,176/36,315)	39% (749/1,910)	
Distance to nearest high-level facility (km)	7.65 (4.3)	8.10 (4.16)	0.058
Antenatal care			
< 4 visits	17% (6,373/37,104)	17% (330/1,927)	0.762
4 - 8 visits	76% (28,253/37,104)	76% (1,461/1,927)	
8 or more visits	7% (2,478/37,104)	7% (136/1,927)	
Multiple gestation	2% (573/37,124)	5% (95/1,928)	< 0.001
Previous caesarean	5% (1,677/37,119)	11% (205/1,928)	< 0.001
Previous miscarriage	16% (5,769/37,120)	17% (331/1,927)	0.066
Gestational complications	7% (2,746/36,669)	8% (152/1,928)	0.560

Intrapartum travel distance (km)	16.83 (19.06)	33.82 (28.13)	< 0.001

Appendix 2 legend: Results reported as percentage who reported affirmative response (number of affirmative responses / total number of non-missing responses) or mean (standard deviation). p-values are calculated from ordinal logistic regression models or linear regression models, both accounting for standard errors at the enrollment facility-level, where applicable. Abbreviations: km = kilometers; UMFL = upward movement in facility level

Appendix 3. Demographic and obstetrics characteristics by availability of full geospatial data.

Characteristic	No geodata (N = 20,642)	Geodata (N = 40,304)	p-value
Age			
19 years or younger	19% (3,989/20,642)	19% (7,585/40,304)	0.522
20 - 34 years	71% (14,752/20,642)	72% (29,093/40,304)	
Older than 34 years	9% (1,901/20,642)	9% (3,626/40,304)	
Education			
Did not complete primary	18% (3,406/18,523)	16% (6,385/39,450)	< 0.001
Completed at least primary	46% (8,563/18,523)	45% (17,616/39,450)	
Completed at least secondary	35% (6,554/18,523)	39% (15,449/39,450)	
Antenatal care			
< 4 visits	19% (3,868/20,307)	17% (6,945/40,281)	< 0.001
4 - 8 visits	75% (15,192/20,307)	76% (30,628/40,281)	
8 or more visits	6% (1,247/20,307)	7% (2,708/40,281)	
Multiple gestation	2% (327/20,261)	2% (689/40,304)	0.314
Previous caesarean	4% (923/20,636)	5% (1,965/40,299)	0.020
Previous miscarriage	15% (3,165/20,638)	16% (6,325/40,299)	0.251
Gestational complications	7% (1,359/18,726)	8% (3,000/39,842)	0.389

Appendix 3 legend: Results reported as percentage who reported affirmative response (number of affirmative responses / total number of non-missing responses) or mean (standard deviation). p-values are calculated from

ordinal logistic regression models or linear regression models, both accounting for standard errors at the enrollment facility-level, where applicable.

Appendix 4. Univariable and fully adjusted logistic regression models estimating the relationship between the likelihood of an adverse health outcome and selected risk factors.

	Unadjusted		Adjusted	
**Characteristic**	**Odds ratio (95% CI)**	**p - value**	**Odds ratio (95% CI)**	**p - value**
UMFL	2.77 (2.17 - 3.54)	< 0.001	2.36 (1.76 - 3.17)	< 0.001
Age				
20 - 34 years	0.89 (0.73 - 1.08)	0.230	0.79 (0.65 - 0.97)	0.022
35 years or older	1.09 (0.84 - 1.43)	0.503	0.93 (0.70 - 1.23)	0.603
Education				
Completed at least primary	0.98 (0.80 - 1.20)	0.836	0.99 (0.81 - 1.20)	0.916
Completed at least secondary	1.03 (0.85 - 1.26)	0.748	1.10 (0.91 - 1.33)	0.325
Distance to nearest high-level facility (km)	1.00 (0.98 - 1.01)	0.556	0.99 (0.98 - 1.01)	0.288
Antenatal care				
4 - 7 visits	0.80 (0.66 - 0.96)	0.017	0.80 (0.66 - 0.96)	0.019
8 or more visits	0.78 (0.55 - 1.09)	0.143	0.78 (0.55 - 1.11)	0.165
Multiple gestation	9.40 (7.67 - 11.53)	< 0.001	8.72 (6.93 - 10.97)	< 0.001
Previous caesarean	1.45 (1.05 - 2.00)	0.022	1.40 (0.97 - 2.01)	0.073
Previous miscarriage	1.11 (0.91 - 1.36)	0.291	1.10 (0.89 - 1.35)	0.370
Gestational complications	1.16 (0.90 - 1.5)	0.261	1.09 (0.85 - 1.40)	0.514

Appendix 4 legend: All models estimated with standard errors adjusted for clustering at the enrollment antenatal facility. Gestational complications include diagnosis of anemia, hypertension, and/or diabetes during pregnancy. Abbreviations: km = kilometers; UMFL = upward movement in facility level

Appendix 5. Kernel-weighted local polynomial estimation of the relationship between total travel distance and probability of adverse health outcome among those who experienced UMFL during delivery episode.

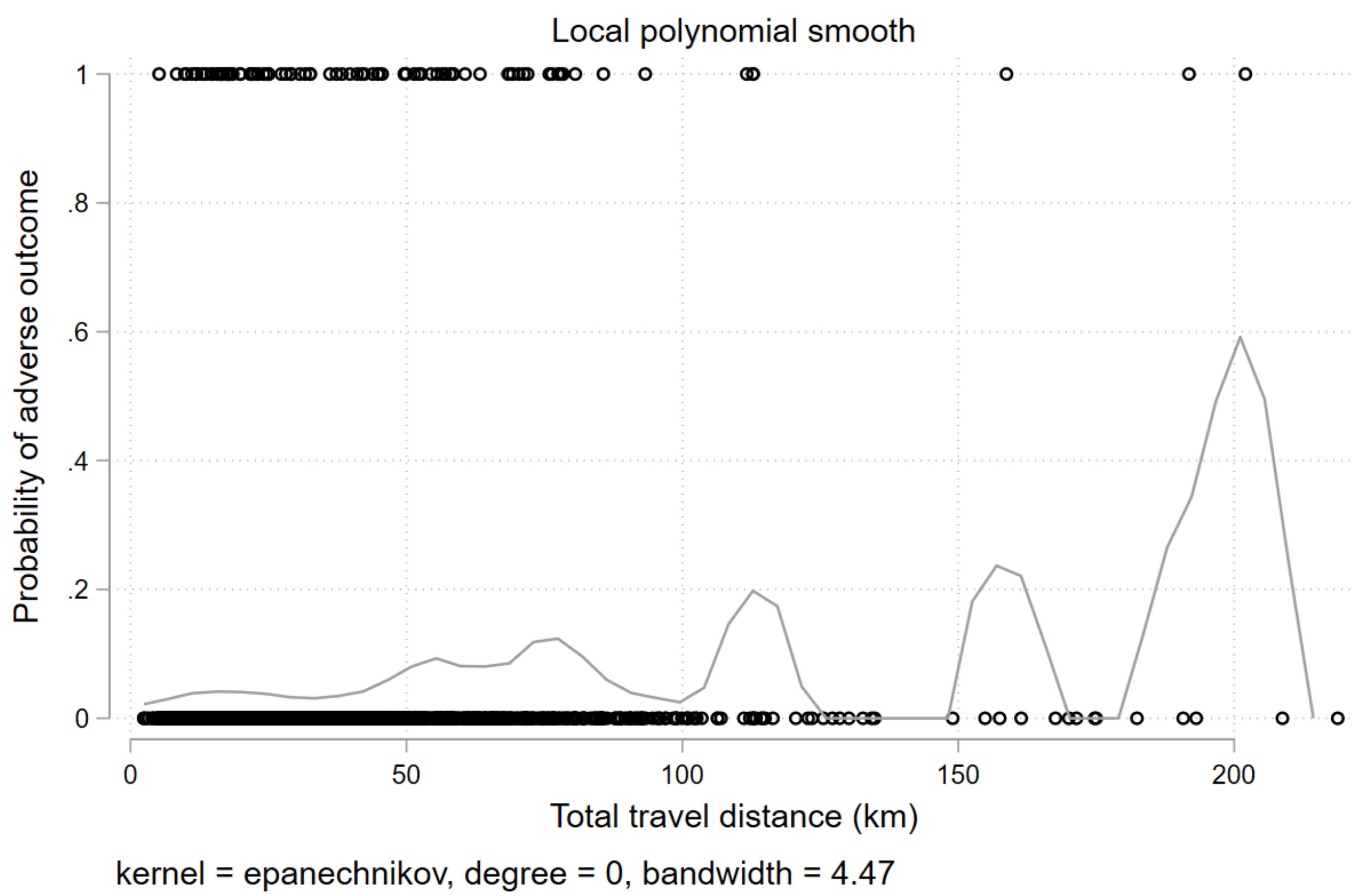

Appendix 5 legend: Omitted one observation with total travel distance of 435.7 km due to data sparsity. Abbreviations: km = kilometers; UMFL = upward movement in facility level.

Appendix 6. Number of events in each quartile of total travel distance and in total among those who experienced UMFL during delivery episode.

	1st Quartile (N = 484)	2nd Quartile (N = 480)	3rd Quartile (N = 486)	4th Quartile (N = 478)	Total
**Stillbirth**	1	5	1	5	12
**Neonatal mortality**	16	17	14	38	85
**Maternal mortality**	0	0	0	1	1
**Any adverse outcome**	17	21	14	40	92

Appendix 6 legend: Quartile 1 = [2.4 km – 16.4 km]; Quartile 2 = [16.4 km – 25.3 km]; Quartile 3 = [25.3 km – 42.3 km]; Quartile 4 = [42.3 km – 435.7 km]. Abbreviations: UMFL = upward movement in facility level.

Appendix 7. Logistic regressions estimating relationship between total travel distance during delivery episode categorized in quartiles and the likelihood of an adverse health outcome among those who experienced UMFL during delivery episode

Characteristic	(1) Odds ratio (95% CI)	(1) p - value	(2) Odds ratio (95% CI)	(2) p - value	(3) Odds ratio (95% CI)	(3) p - value
Travel distance quartile						
2nd quartile	1.26 (0.64 - 2.47)	0.506	1.30 (0.64 - 2.61)	0.467	1.51 (0.70 - 3.27)	0.294
3rd quartile	0.81 (0.43 - 1.56)	0.537	0.83 (0.43 - 1.60)	0.584	0.92 (0.47 - 1.80)	0.817
4th quartile	2.51 (1.47 - 4.29)	0.001	2.49 (1.42 - 4.37)	0.001	2.60 (1.43 - 4.74)	0.002
Age						
20 - 34 years			0.93 (0.55 - 1.58)	0.801	1.02 (0.59 - 1.77)	0.932
35 years or older			1.33 (0.61 - 2.90)	0.473	1.14 (0.51 - 2.53)	0.748
Education						
Completed at least primary			0.58 (0.36 - 0.93)	0.024	0.58 (0.35 - 0.94)	0.026
Completed at least secondary			0.61 (0.35 - 1.06)	0.081	0.59 (0.33 - 1.05)	0.075
Distance to nearest high-level facility (km)					0.97 (0.92 - 1.02)	0.253
Antenatal care						
4 - 7 visits					0.72 (0.48 - 1.07)	0.107
8 or more visits					0.56 (0.21 - 1.51)	0.253
Multiple gestation					4.64 (2.53 - 8.53)	< 0.001
Previous caesarean					0.17 (0.04 - 0.74)	0.019
Previous miscarriage					0.91 (0.49 - 1.71)	0.769

Gestational complications	1.64<br>(0.95 - 2.82)	0.076

Appendix 7 legend: Column (1) presents results from unadjusted model, column (2) presents results from model adjusted for demographic characteristics, and column (3) presents results from model adjusted for demographic and obstetric characteristics. All models estimated with standard errors adjusted for clustering at the enrollment antenatal facility. Gestational complications include diagnosis of anemia, hypertension, and/or diabetes during pregnancy. Travel quartile ranges: Quartile 1 = [2.4 km – 16.4 km]; Quartile 2 = [16.4 km – 25.3 km]; Quartile 3 = [25.3 km – 42.3 km]; Quartile 4 = [42.3 km – 435.7 km]. Abbreviations: km = kilometers; UMFL = upward movement in facility level

Appendix 8. Logistic regressions estimating relationship between total travel distance during delivery episode categorized in quartiles and the likelihood of an adverse health outcome among those who visited multiple facilities during intrapartum.

	(1)		(2)		(3)	
**Characteristic**	**Odds ratio (95% CI)**	**p - value**	**Odds ratio (95% CI)**	**p - value**	**Odds ratio (95% CI)**	**p - value**
Travel distance quartile						
2nd quartile	1.20 (0.75 - 1.92)	0.439	1.22 (0.76 - 1.96)	0.405	1.27 (0.78 - 2.06)	0.338
3rd quartile	1.86 (1.16 – 3.00)	0.010	1.72 (1.05 - 2.80)	0.030	1.78 (1.11 - 2.87)	0.017
4th quartile	3.23 (2.15 - 4.85)	< 0.001	3.16 (2.09 - 4.78)	< 0.001	3.14 (2.04 - 4.83)	< 0.001
Age						
20 - 34 years			1.39 (0.96 - 2.01)	0.085	1.44 (1.00 - 2.06)	0.048
35 years or older			1.38 (0.83 - 2.29)	0.219	1.14 (0.65 – 2.00)	0.652
Education						
Completed at least primary			0.80 (0.56 - 1.15)	0.225	0.80 (0.55 - 1.17)	0.251
Completed at least secondary			0.72 (0.49 - 1.06)	0.100	0.71 (0.46 - 1.08)	0.106
Distance to nearest high-level facility (km)					0.98 (0.94 - 1.02)	0.384
Antenatal care						
4 - 7 visits					0.65 (0.44 - 0.94)	0.022
8 or more visits					0.41 (0.21 - 0.82)	0.012
Multiple gestation					4.04 (2.68 - 6.07)	< 0.001
Previous caesarean					0.66 (0.39 - 1.11)	0.114
Previous miscarriage					1.22 (0.78 - 1.89)	0.386

Gestational complications	1.33<br>(0.85 - 2.09)	0.210

Appendix 8 legend: Column (1) presents results from unadjusted model, column (2) presents results from model adjusted for demographic characteristics, and column (3) presents results from model adjusted for demographic and obstetric characteristics. All models estimated with standard errors adjusted for clustering at the enrollment antenatal facility. Gestational complications include diagnosis of anemia, hypertension, and/or diabetes during pregnancy Travel quartile ranges: Quartile 1 = [2.3 km – 17.3 km]; Quartile 2 = [17.4 km – 26.9 km]; Quartile 3 = [27.0 km – 44.9 km]; Quartile 4 = [44.9 km – 435.7 km]. Abbreviations: km = kilometers; UMFL = upward movement in facility level.

Appendix 9. Logistic regressions estimating relationship between total travel distance during delivery episode categorized in quartiles and the likelihood of an adverse health outcome among those who visited multiple facilities during intrapartum including moves from high-level facilities to low-level facilities.

Characteristic	(1) Odds ratio (95% CI)	(1) p - value	(2) Odds ratio (95% CI)	(2) p - value	(3) Odds ratio (95% CI)	(3) p - value
Travel distance quartile						
2nd quartile	1.20 (0.76 - 1.90)	0.432	1.22 (0.77 - 1.93)	0.402	1.26 (0.78 - 2.03)	0.349
3rd quartile	1.98 (1.23 - 3.18)	0.005	1.83 (1.14 - 2.95)	0.013	1.90 (1.19 - 3.03)	0.007
4th quartile	3.18 (2.10 - 4.79)	< 0.001	3.11 (2.04 - 4.73)	< 0.001	3.03 (1.95 - 4.71)	< 0.001
Age						
20 - 34 years			1.40 (0.97 - 2.01)	0.069	1.43 (1.01 - 2.02)	0.046
35 years or older			1.36 (0.81 - 2.26)	0.243	1.12 (0.64 - 1.97)	0.68
Education						
Completed at least primary			0.79 (0.55 - 1.14)	0.205	0.79 (0.54 - 1.15)	0.223
Completed at least secondary			0.74 (0.50 - 1.09)	0.130	0.72 (0.47 - 1.09)	0.123
Distance to nearest high-level facility (km)					0.98 (0.95 - 1.02)	0.402
Antenatal care						
4 - 7 visits					0.64 (0.44 - .92)	0.017
8 or more visits					0.46 (0.24 - 0.91)	0.025
Multiple gestation					4.05 (2.68 - 6.13)	< 0.001
Previous caesarean					0.67 (0.40 - 1.14)	0.138

Previous miscarriage	1.21<br>(0.78 - 1.88)	0.387
Gestational complications	1.30<br>(0.83 - 2.04)	0.245

Appendix 9 legend: Column (1) presents results from unadjusted model, column (2) presents results from model adjusted for demographic characteristics, and column (3) presents results from model adjusted for demographic and obstetric characteristics. All models estimated with standard errors adjusted for clustering at the enrollment antenatal facility. Gestational complications include diagnosis of anemia, hypertension, and/or diabetes during pregnancy. Travel quartile ranges: Quartile 1 = [1.0 km – 17.2 km]; Quartile 2 = [17.2 km – 26.9 km]; Quartile 3 = [26.9 km – 45.9 km]; Quartile 4 = [44.9 km – 435.7 km]. Abbreviations: km = kilometers; UMFL = upward movement in facility level.

Appendix 10. Logistic regressions estimating relationship between total travel distance during delivery episode categorized in quartiles and the likelihood of an adverse health outcome among those who experienced UMFL during delivery episode, without restricting based on home village being within Kakamega County or first leg of journey

	(1)		(2)		(3)	
**Characteristic**	**Odds ratio (95% CI)**	**p - value**	**Odds ratio (95% CI)**	**p - value**	**Odds ratio (95% CI)**	**p - value**
Travel distance quartile						
2nd quartile	1.26 (0.64 - 2.47)	0.509	1.30 (0.64 - 2.62)	0.471	1.51 (0.70 - 3.27)	0.294
3rd quartile	0.88 (0.50 - 1.56)	0.672	0.84 (0.44 - 1.6)	0.595	0.93 (0.48 - 1.80)	0.837
4th quartile	2.62 (1.50 - 4.59)	0.001	2.69 (1.53 - 4.73)	0.001	2.79 (1.54 - 5.07)	0.001
Age						
20 - 34 years			0.98 (0.58 - 1.66)	0.948	1.04 (0.61 - 1.79)	0.875
35 years or older			1.50 (0.70 - 3.18)	0.295	1.30 (0.60 - 2.81)	0.500
Education						
Completed at least primary			0.61 (0.38 - 0.98)	0.039	0.60 (0.37 - 0.97)	0.039
Completed at least secondary			0.63 (0.36 - 1.09)	0.101	0.62 (0.34 - 1.12)	0.112
Distance to nearest high-level facility (km)					0.97 (0.92 - 1.02)	0.203
Antenatal care						
4 - 7 visits					0.75 (0.51 - 1.1)	0.138
8 or more visits					0.55 (0.20 - 1.5)	0.243
Multiple gestation					4.69 (2.57 - 8.58)	< 0.001
Previous caesarean					0.17	0.017

	(0.04 - .73)	
Previous miscarriage	0.91<br>(0.50 - 1.68)	0.772
Gestational complications	1.56<br>(0.90 - 2.71)	0.110

Appendix 10 legend: Column (1) presents results from unadjusted model, column (2) presents results from model adjusted for demographic characteristics, and column (3) presents results from model adjusted for demographic and obstetric characteristics. All models estimated with standard errors adjusted for clustering at the enrollment antenatal facility. Gestational complications include diagnosis of anemia, hypertension, and/or diabetes during pregnancy. Travel quartile ranges: Quartile 1 = [2.4 km – 16.4 km]; Quartile 2 = [16.4 km – 25.4 km]; Quartile 3 = [25.4 km – 42.8 km]; Quartile 4 = [42.8 km – 435.7 km]. Abbreviations: km = kilometers; UMFL = upward movement in facility level .

Appendix 11. Logistic regressions estimating relationship between total travel distance during delivery episode categorized in quartiles and the likelihood of an adverse health outcome among those who experienced UMFL during delivery episode and whose final facility was Kakamega County General Hospital.

Characteristic	(1) Odds ratio (95% CI)	(1) p - value	(2) Odds ratio (95% CI)	(2) p - value	(3) Odds ratio (95% CI)	(3) p - value
Travel distance quartile						
2nd quartile	1.21 (0.38 - 3.84)	0.746	1.24 (0.39 - 3.99)	0.716	1.24 (0.36 - 4.27)	0.738
3rd quartile	2.28 (0.89 - 5.8)	0.084	2.45 (0.94 - 6.39)	0.066	2.43 (0.90 - 6.57)	0.080
4th quartile	5.24 (2.21 - 12.45)	< 0.001	5.48 (2.12 - 14.15)	< 0.001	4.27 (1.38 - 13.25)	0.012
Age						
20 - 34 years			1.15 (0.51 - 2.61)	0.737	1.34 (0.56 - 3.21)	0.513
35 years or older			1.84 (0.59 - 5.74)	0.296	1.27 (0.34 - 4.76)	0.721
Education						
Completed at least primary			0.33 (0.17 - 0.64)	0.001	0.33 (0.17 - 0.64)	0.001
Completed at least secondary			0.29 (0.11 - 0.78)	0.014	0.26 (0.09 - 0.75)	0.012
Distance to nearest high-level facility (km)					0.92 (0.83 - 1.03)	0.158
Antenatal care						
4 - 7 visits					0.62 (0.38 - 1.04)	0.068
8 or more visits					0.35 (0.11 - 1.12)	0.078

Multiple gestation	3.57 (1.30 - 9.79)	0.013
Previous caesarean	OMITTED	-
Previous miscarriage	1.52 (0.65 - 3.57)	0.336
Gestational complications	1.93 (0.87 - 4.27)	0.105

Appendix 11 legend: Column (1) presents results from unadjusted model, column (2) presents results from model adjusted for demographic characteristics, and column (3) presents results from model adjusted for demographic and obstetric characteristics. All models estimated with standard errors adjusted for clustering at the enrollment antenatal facility. Gestational complications include diagnosis of anemia, hypertension, and/or diabetes during pregnancy. Previous caesarean omitted due to collinearity in column (3). Travel quartile ranges: Quartile 1 = [10.7 km – 22.1 km]; Quartile 2 = [22.4 km – 35.3 km]; Quartile 3 = [35.4 km – 56.2 km]; Quartile 4 = [56.4 km – 171.4 km]. Abbreviations: km = kilometers; UMFL = upward movement in facility level.

Appendix 12. Logistic regressions estimating relationship between total travel distance during delivery episode categorized in quartiles and the likelihood of an adverse health outcome among those who experienced UMFL during delivery episode and whose final facility was level 5 or 6.

Characteristic	(1) Odds ratio (95% CI)	(1) p - value	(2) Odds ratio (95% CI)	(2) p - value	(3) Odds ratio (95% CI)	(3) p - value
Travel distance quartile						
2nd quartile	1.44 (0.48 - 4.31)	0.519	1.51 (0.49 - 4.68)	0.471	1.44 (0.44 - 4.78)	0.547
3rd quartile	2.55 (0.98 - 6.63)	0.055	2.73 (1.02 - 7.32)	0.046	2.48 (0.90 - 6.8)	0.078
4th quartile	4.93 (2.01 - 12.05)	< 0.001	5.25 (1.95 - 14.11)	0.001	4.07 (1.26 - 13.19)	0.019
Age						
20 - 34 years			1.14 (0.51 - 2.56)	0.745	1.36 (0.58 - 3.19)	0.486
35 years or older			1.81 (0.58 - 5.61)	0.303	1.27 (0.34 - 4.71)	0.719
Education						
Completed at least primary			0.31 (0.16 - 0.61)	0.001	0.31 (0.16 - .62)	0.001
Completed at least secondary			0.30 (0.12 - 0.78)	0.014	0.28 (0.10 - .76)	0.013
Distance to nearest high-level facility (km)					0.92 (0.83 - 1.03)	0.141
Antenatal care						
4 - 7 visits					0.55 (0.33 - .92)	0.021
8 or more visits					0.32 (0.10 - 1.04)	0.058
Multiple gestation					3.40 (1.22 - 9.46)	0.019

Previous caesarean	OMITTED	-
Previous miscarriage	1.49<br>(0.64 - 3.43)	0.353
Gestational complications	1.87<br>(0.85 - 4.11)	0.120

Appendix 12 legend: Column (1) presents results from unadjusted model, column (2) presents results from model adjusted for demographic characteristics, and column (3) presents results from model adjusted for demographic and obstetric characteristics. All models estimated with standard errors adjusted for clustering at the enrollment antenatal facility. Gestational complications include diagnosis of anemia, hypertension, and/or diabetes during pregnancy. Previous caesarean omitted due to collinearity in column (3). Travel quartile ranges: Quartile 1 = [10.7 km – 22.7 km]; Quartile 2 = [22.7 km – 36.1 km]; Quartile 3 = [36.1 km – 57.0 km]; Quartile 4 = [57.1 km – 208.7 km]. Abbreviations: km = kilometers; UMFL = upward movement in facility level.

Appendix 13. Logistic regressions estimating relationship between total travel distance during delivery episode categorized in quartiles and the likelihood of an adverse health outcome among those who experienced UMFL during delivery episode and excluding those living in Butere, Lugari, and Malava.

Characteristic	(1) Odds ratio (95% CI)	(1) p - value	(2) Odds ratio (95% CI)	(2) p - value	(3) Odds ratio (95% CI)	(3) p - value
Travel distance quartile						
2nd quartile	1.00 (0.39 - 2.55)	0.994	1.03 (0.39 - 2.70)	0.948	1.17 (0.42 - 3.27)	0.765
3rd quartile	0.79 (0.38 - 1.68)	0.547	0.80 (0.38 - 1.67)	0.545	0.92 (0.43 – 2.00)	0.843
4th quartile	2.53 (1.32 - 4.86)	0.005	2.56 (1.34 - 4.91)	0.005	2.71 (1.37 - 5.36)	0.004
Age						
20 - 34 years			1.10 (0.61 – 2.00)	0.745	1.25 (0.68 - 2.29)	0.481
35 years or older			1.45 (0.43 - 4.93)	0.549	1.20 (0.32 - 4.53)	0.786
Education						
Completed at least primary			0.71 (0.35 - 1.43)	0.340	0.72 (0.36 - 1.45)	0.356
Completed at least secondary			0.74 (0.35 - 1.55)	0.423	0.74 (0.36 - 1.53)	0.419
Distance to nearest high-level facility (km)					1.01 (0.95 - 1.07)	0.787
Antenatal care						
4 - 7 visits					0.54 (0.36 - 0.81)	0.003
8 or more visits					0.43 (0.13 - 1.39)	0.159
Multiple gestation					4.42 (2.14 - 9.13)	< 0.001

Previous caesarean	0.14<br>(0.02 - 1.19)	0.072
Previous miscarriage	1.42<br>(0.75 - 2.66)	0.281
Gestational complications	1.84<br>(1.01 - 3.33)	0.045

Appendix 13 legend: Column (1) presents results from unadjusted model, column (2) presents results from model adjusted for demographic characteristics, and column (3) presents results from model adjusted for demographic and obstetric characteristics. All models estimated with standard errors adjusted for clustering at the enrollment antenatal facility. Gestational complications include diagnosis of anemia, hypertension, and/or diabetes during pregnancy. Previous caesarean omitted due to collinearity in column (3). Travel quartile ranges: Quartile 1 = [2.4 km – 16.7 km]; Quartile 2 = [16.7 km – 26.8 km]; Quartile 3 = [26.8 km – 42.3 km]; Quartile 4 = [42.3 km – 435.7 km]. Abbreviations: km = kilometers; UMFL = upward movement in facility level.

Appendix 14. Table of estimates of odds ratios associated with each quartile across the main model and six highlighted sensitivity analyses.

	Quartile 2		Quartile 3		Quartile 4	
	**Odds Ratio (95% CI)**	**% change from Main**	**Odds Ratio (95% CI)**	**% change from Main**	**Odds Ratio (95% CI)**	**% change from Main**
**Main**	1.51 (0.70 - 3.27)	100%	0.92 (0.47 - 1.80)	100%	2.60 (1.43 - 4.74)	100%
**Sensitivity 1**	1.27 (0.78 - 2.06)	84%	1.78 (1.11 - 2.87)	193%	3.14 (2.04 - 4.83)	121%
**Sensitivity 2**	1.26 (0.78 - 2.03)	83%	1.90 (1.19 - 3.03)	207%	3.03 (1.95 - 4.71)	117%
**Sensitivity 3**	1.51 (0.70 - 3.27)	100%	0.93 (0.48 - 1.8)	101%	2.79 (1.54 - 5.07)	107%
**Sensitivity 4**	1.24 (0.36 - 4.27)	82%	2.43 (0.90 - 6.57)	264%	4.27 (1.38 - 13.25)	164%
**Sensitivity 5**	1.44 (0.44 - 4.78)	95%	2.48 (0.90 - 6.80)	270%	4.07 (1.26 - 13.19)	157%
**Sensitivity 6**	1.17 (0.42 - 3.27)	77%	0.92 (0.43 – 2.00)	100%	2.71 (1.37 - 5.36)	104%

Appendix 14 legend: Results from models adjusted for demographic and obstetric characteristics. All models estimated with bootstrapped standard errors adjusted for clustering at the enrollment antenatal facility. Reference category: 1st quartile. Percentage change calculated by dividing the estimated odds ratio for each quartile and model by the respective estimated odds ration for that quartile from the main model and then multiplying by 100. Sensitivity Model 1: sample was restricted to those who reported multiple facilities during intrapartum. Sensitivity Model 2: sample was restricted to those who reported multiple facilities during intrapartum and the assumption that low-level facilities reported after high-level facilities were reporting errors was relaxed. Sensitivity Model 3: sample was no longer restricted to only those who reported living in Kakamega County and whose first leg of their delivery journey was equal to or less than 111 km. Sensitivity Model 4: sample was restricted to those who visited Kakamega County General Hospital as their final facility. Sensitivity Model 5: sample was restricted to those whose final

facility reported was level 5 or 6. Sensitivity Model 6: sample excludes those who reported living in those sub-counties that were designated to receive the health system intervention as a part of Service Delivery Redesign for Maternal and Newborn Health.

Appendix 15. Logistic regressions estimating relationship between total travel distance during delivery episode categorized in quartiles and the likelihood of stillbirth among those who experienced UMFL during delivery episode.

	(1)		(2)		(3)	
**Characteristic**	**Odds ratio (95% CI)**	**p - value**	**Odds ratio (95% CI)**	**p - value**	**Odds ratio (95% CI)**	**p - value**
Travel distance quartile						
2nd quartile	5.08 (0.54 - 47.43)	0.154	5.55 (0.58 - 53.4)	0.138	7.14 (0.54 - 94.42)	0.136
3rd quartile	1.00 (0.06 - 16.96)	0.998	1.08 (0.06 - 18.86)	0.958	1.29 (0.06 - 28.68)	0.873
4th quartile	5.11 (0.54 - 47.99)	0.154	5.39 (0.56 - 51.73)	0.144	6.03 (0.52 - 69.91)	0.151
Age						
20 - 34 years			1.29 (0.24 - 6.97)	0.770	0.98 (0.17 - 5.67)	0.980
35 years or older			1.81 (0.21 - 15.79)	0.593	0.70 (0.10 - 4.79)	0.712
Education						
Completed at least primary			0.41 (0.14 - 1.23)	0.113	0.40 (0.12 - 1.33)	0.135
Completed at least secondary			0.29 (0.07 - 1.24)	0.094	0.34 (0.09 - 1.27)	0.108
Distance to nearest high-level facility (km)					1.00 (0.90 - 1.11)	0.984
Antenatal care						
4 - 7 visits					1.34 (0.31 - 5.87)	0.695
8 or more visits					4.51 (0.62 - 32.87)	0.137
Multiple gestation					24.57 (4.94 - 122.23)	< 0.001

Previous caesarean	OMITTED	-
Previous miscarriage	1.27<br>(0.42 - 3.85)	0.670
Gestational complications	4.34<br>(0.70 - 27.07)	0.116

Appendix 15 legend: Column (1) presents results from unadjusted model, column (2) presents results from model adjusted for demographic characteristics, and column (3) presents results from model adjusted for demographic and obstetric characteristics. All models estimated with bootstrapped standard errors adjusted for clustering at the enrollment antenatal facility. Gestational complications include diagnosis of anemia, hypertension, and/or diabetes during pregnancy. Travel quartile ranges: Quartile 1 = [2.4 km – 16.4 km]; Quartile 2 = [16.4 km – 25.3 km]; Quartile 3 = [25.3 km – 42.3 km]; Quartile 4 = [42.3 km – 435.7 km]. Abbreviations: km = kilometers; UMFL = upward movement in facility level

Appendix 16. Logistic regressions estimating relationship between total travel distance during delivery episode categorized in quartiles and the likelihood of neonatal mortality among those who experienced UMFL during delivery episode.

Characteristic	(1) Odds ratio (95% CI)	(1) p - value	(2) Odds ratio (95% CI)	(2) p - value	(3) Odds ratio (95% CI)	(3) p - value
Travel distance quartile						
2nd quartile	1.08 (0.55 - 2.11)	0.825	1.12 (0.56 - 2.25)	0.757	1.32 (0.61 - 2.83)	0.478
3rd quartile	0.86 (0.43 - 1.75)	0.685	0.89 (0.43 - 1.81)	0.737	0.99 (0.48 - 2.05)	0.977
4th quartile	2.53 (1.43 - 4.48)	0.001	2.51 (1.37 - 4.57)	0.003	2.71 (1.45 - 5.06)	0.002
Age						
20 - 34 years			0.91 (0.53 - 1.56)	0.735	0.99 (0.56 - 1.73)	0.963
35 years or older			1.28 (0.57 - 2.85)	0.553	1.22 (0.54 - 2.74)	0.632
Education						
Completed at least primary			0.56 (0.35 - .92)	0.023	0.57 (0.34 - .96)	0.035
Completed at least secondary			0.58 (0.34 - 1.01)	0.056	0.61 (0.33 - 1.1)	0.101
Distance to nearest high-level facility (km)					0.96 (0.91 - 1.02)	0.172
Antenatal care						
4 - 7 visits					0.66 (0.44 – 1.00)	0.051
8 or more visits					0.44 (0.15 - 1.30)	0.137
Multiple gestation					5.04 (2.72 - 9.34)	< 0.001
Previous caesarean					0.19 (0.04 - 0.81)	0.025

Previous miscarriage	0.92<br>(0.49 - 1.75)	0.808
Gestational complications	1.40<br>(0.79 - 2.47)	0.244

Appendix 16 legend: Column (1) presents results from unadjusted model, column (2) presents results from model adjusted for demographic characteristics, and column (3) presents results from model adjusted for demographic and obstetric characteristics. All models estimated with bootstrapped standard errors adjusted for clustering at the enrollment antenatal facility. Gestational complications include diagnosis of anemia, hypertension, and/or diabetes during pregnancy. Travel quartile ranges: Quartile 1 = [2.4 km – 16.4 km]; Quartile 2 = [16.4 km – 25.3 km]; Quartile 3 = [25.3 km – 42.3 km]; Quartile 4 = [42.3 km – 435.7 km]. Abbreviations: km = kilometers; UMFL = upward movement in facility level

Appendix 17. Firth logistic regressions estimating relationship between total travel distance during delivery episode categorized in quartiles and the likelihood of an adverse health outcome among those who experienced UMFL during delivery episode

Characteristic	(1) Odds ratio (95% CI)	(1) p - value	(2) Odds ratio (95% CI)	(2) p - value	(3) Odds ratio (95% CI)	(3) p - value
Travel distance quartile						
2nd quartile	1.25 (0.59 - 2.63)	0.557	1.29 (0.69 - 2.42)	0.429	1.49 (0.69 - 3.25)	0.311
3rd quartile	0.82 (0.41 - 1.65)	0.579	0.84 (0.42 - 1.66)	0.612	0.93 (0.49 - 1.78)	0.832
4th quartile	2.47 (1.33 - 4.57)	0.004	2.44 (1.44 - 4.16)	0.001	2.54 (1.49 - 4.32)	0.001
Age						
20 - 34 years			0.92 (0.55 - 1.55)	0.756	1.01 (0.58 - 1.75)	0.972
35 years or older			1.35 (0.64 - 2.84)	0.436	1.15 (0.43 - 3.10)	0.778
Education						
Completed at least primary			0.58 (0.37 - 0.91)	0.018	0.57 (0.31 - 1.07)	0.080
Completed at least secondary			0.61 (0.36 - 1.03)	0.065	0.59 (0.32 - 1.09)	0.092
Distance to nearest high-level facility (km)					0.97 (0.93 - 1.02)	0.250
Antenatal care						
4 - 7 visits					0.71 (0.47 - 1.09)	0.116
8 or more visits					0.61 (0.22 - 1.70)	0.342
Multiple gestation					4.60 (2.47 - 8.57)	< 0.001
Previous caesarean					0.21 (0.05 - 0.87)	0.031
Previous miscarriage					0.93	0.803

	(0.52 - 1.65)	
Gestational complications	1.68<br>(0.94 - 3.01)	0.081

Appendix 17 legend: Column (1) presents results from unadjusted model, column (2) presents results from model adjusted for demographic characteristics, and column (3) presents results from model adjusted for demographic and obstetric characteristics. All models estimated with bootstrapped standard errors adjusted for clustering at the enrollment antenatal facility. Gestational complications include diagnosis of anemia, hypertension, and/or diabetes during pregnancy. Travel quartile ranges: Quartile 1 = [2.4 km – 16.4 km]; Quartile 2 = [16.4 km – 25.3 km]; Quartile 3 = [25.3 km – 42.3 km]; Quartile 4 = [42.3 km – 435.7 km]. Abbreviations: km = kilometers; UMFL = upward movement in facility level

Appendix 18. Logistic regressions estimating relationship between total travel distance during delivery episode categorized in quartiles and the likelihood of an adverse health outcome among those who experienced UMFL during delivery episode, omitting extreme travel distance data point (435.7 km).

Characteristic	(1) Odds ratio (95% CI)	(1) p - value	(2) Odds ratio (95% CI)	(2) p - value	(3) Odds ratio (95% CI)	(3) p - value
Travel distance quartile						
2nd quartile	1.26 (0.64 - 2.47)	0.506	1.30 (0.64 - 2.62)	0.466	1.51 (0.70 - 3.27)	0.294
3rd quartile	0.81 (0.43 - 1.56)	0.537	0.83 (0.43 - 1.61)	0.585	0.92 (0.47 - 1.80)	0.818
4th quartile	2.51 (1.47 - 4.30)	0.001	2.50 (1.42 - 4.39)	0.001	2.61 (1.43 - 4.76)	0.002
Age						
20 - 34 years			0.94 (0.55 - 1.59)	0.807	1.03 (0.59 - 1.77)	0.925
35 years or older			1.33 (0.61 - 2.90)	0.476	1.14 (0.51 - 2.52)	0.751
Education						
Completed at least primary			0.58 (0.36 - 0.92)	0.022	0.57 (0.35 - 0.93)	0.025
Completed at least secondary			0.61 (0.35 - 1.05)	0.076	0.58 (0.32 - 1.05)	0.071
Distance to nearest high-level facility (km)					0.97 (0.92 - 1.02)	0.250
Antenatal care						
4 - 7 visits					0.72 (0.48 - 1.08)	0.110
8 or more visits					0.56 (0.21 - 1.51)	0.253
Multiple gestation					4.64 (2.53 - 8.51)	< 0.001
Previous caesarean					0.17 (0.04 - 0.74)	0.019

Previous miscarriage	0.91 (0.48 - 1.70)	0.764
Gestational complications	1.64 (0.95 - 2.82)	0.077

Appendix 18 legend: Column (1) presents results from unadjusted model, column (2) presents results from model adjusted for demographic characteristics, and column (3) presents results from model adjusted for demographic and obstetric characteristics. All models estimated with standard errors adjusted for clustering at the enrollment antenatal facility. Gestational complications include diagnosis of anemia, hypertension, and/or diabetes during pregnancy. Travel quartile ranges: Quartile 1 = [2.4 km – 16.4 km]; Quartile 2 = [16.4 km – 25.3 km]; Quartile 3 = [25.3 km – 42.3 km]; Quartile 4 = [42.3 km – 218.8 km]. Abbreviations: km = kilometers; UMFL = upward movement in facility level

Appendix 19. Logistic regressions estimating relationship between total travel distance during delivery episode and the likelihood of an adverse health outcome among those who experienced UMFL during delivery episode).

Characteristic	(1) Odds ratio (95% CI)	(1) p - value	(2) Odds ratio (95% CI)	(2) p - value	(3) Odds ratio (95% CI)	(3) p - value
Travel distance						
15 - 30 km	1.29 (0.68 - 2.45)	0.435	1.34 (0.68 - 2.62)	0.393	1.47 (0.71 - 3.03)	0.301
30 - 60 km	1.65 (0.92 - 2.99)	0.095	1.63 (0.88 – 3.00)	0.118	1.77 (0.94 - 3.33)	0.078
> 60 km	2.90 (1.55 - 5.43)	0.001	2.93 (1.55 - 5.53)	0.001	2.98 (1.53 - 5.80)	0.001
Age						
20 - 34 years			0.92 (0.54 - 1.55)	0.748	1.03 (0.60 - 1.78)	0.921
35 years or older			1.26 (0.58 - 2.74)	0.558	1.13 (0.52 - 2.46)	0.752
Education						
Completed at least primary			0.59 (0.37 - 0.95)	0.028	0.59 (0.36 - 0.95)	0.03
Completed at least secondary			0.60 (0.35 - 1.04)	0.071	0.57 (0.32 - 1.02)	0.058
Distance to nearest high-level facility (km)					0.97 (0.93 - 1.02)	0.255
Antenatal care						
4 - 7 visits					0.70 (0.47 - 1.03)	0.067
8 or more visits					0.56 (0.21 - 1.51)	0.250
Multiple gestation					4.56 (2.50 - 8.31)	< 0.001
Previous caesarean					0.17 (0.04 - 0.73)	0.018
Previous miscarriage					0.93	0.826

	(0.50 - 1.75)	
Gestational complications	1.69<br>(0.99 - 2.88)	0.054

Appendix 19 legend: Column (1) presents results from unadjusted model, column (2) presents results from model adjusted for demographic characteristics, and column (3) presents results from model adjusted for demographic and obstetric characteristics. All models estimated with standard errors adjusted for clustering at the enrollment antenatal facility. Gestational complications include diagnosis of anemia, hypertension, and/or diabetes during pregnancy. Travel distance reference category = “< 15km.” Abbreviations: km = kilometers; UMFL = upward movement in facility level

Appendix 20. Multilevel logistic regressions estimating relationship between total travel distance during delivery episode categorized in quartiles and the likelihood of an adverse health outcome among those who experienced UMFL during delivery episode, with random effects at the ward, subcounty, and first facility levels.

	(1)		(2)		(3)	
**Characteristic**	**Odds ratio (95% CI)**	**p - value**	**Odds ratio (95% CI)**	**p - value**	**Odds ratio (95% CI)**	**p - value**
Travel distance quartile						
2nd quartile	1.26 (0.64 - 2.48)	0.496	1.31 (0.67 - 2.56)	0.438	1.56 (0.77 - 3.16)	0.221
3rd quartile	0.83 (0.40 - 1.74)	0.625	0.85 (0.41 - 1.78)	0.669	0.97 (0.45 - 2.06)	0.929
4th quartile	2.59 (1.42 - 4.73)	0.002	2.57 (1.40 - 4.71)	0.002	2.73 (1.46 - 5.13)	0.002
Age						
20 - 34 years			0.94 (0.54 - 1.63)	0.817	1.03 (0.58 - 1.83)	0.921
35 years or older			1.34 (0.61 - 2.91)	0.464	1.13 (0.48 - 2.63)	0.780
Education						
Completed at least primary			0.58 (0.33 - 1.03)	0.063	0.56 (0.31 - 1.02)	0.059
Completed at least secondary			0.62 (0.34 - 1.12)	0.114	0.58 (0.31 - 1.08)	0.087
Distance to nearest high-level facility (km)					0.97 (0.91 - 1.02)	0.234
Antenatal care						
4 - 7 visits					0.71 (0.42 - 1.22)	0.213
8 or more visits					0.54 (0.19 - 1.53)	0.244
Multiple gestation					4.94 (2.60 - 9.38)	< 0.001
Previous caesarean					0.16 (0.04 - 0.67)	0.012
Previous miscarriage					0.88 (0.48 - 1.62)	0.684

Gestational complications	1.71<br>(0.85 - 3.43)	0.131

Appendix 20 legend: Column (1) presents results from unadjusted model, column (2) presents results from model adjusted for demographic characteristics, and column (3) presents results from model adjusted for demographic and obstetric characteristics. Gestational complications include diagnosis of anemia, hypertension, and/or diabetes during pregnancy. Travel quartile ranges: Quartile 1 = [2.4 km – 16.4 km]; Quartile 2 = [16.4 km – 25.3 km]; Quartile 3 = [25.3 km – 42.3 km]; Quartile 4 = [42.3 km – 435.7 km]. Abbreviations: km = kilometers; UMFL = upward movement in facility level

Appendix 21. Logistic regressions estimating relationship between total travel distance during delivery episode categorized in quartiles and the likelihood of an adverse health outcome among those who experienced UMFL during delivery episode

Characteristic	(1) Odds ratio (95% CI)	(1) p - value	(2) Odds ratio (95% CI)	(2) p - value	(3) Odds ratio (95% CI)	(3) p - value
Travel distance quartile						
2nd quartile	1.26 (0.64 - 2.47)	0.506	1.30 (0.64 - 2.61)	0.467	1.50 (0.69 - 3.28)	0.310
3rd quartile	0.81 (0.43 - 1.56)	0.537	0.83 (0.43 - 1.60)	0.584	0.92 (0.47 - 1.8)	0.812
4th quartile	2.51 (1.47 - 4.29)	0.001	2.49 (1.42 - 4.37)	0.001	2.57 (1.40 - 4.71)	0.002
Age						
20 - 34 years			0.93 (0.55 - 1.58)	0.801	1.02 (0.59 - 1.77)	0.944
35 years or older			1.33 (0.61 - 2.90)	0.473	1.13 (0.51 - 2.51)	0.756
Education						
Completed at least primary			0.58 (0.36 - 0.93)	0.024	0.57 (0.35 - 0.93)	0.024
Completed at least secondary			0.61 (0.35 - 1.06)	0.081	0.58 (0.33 - 1.05)	0.070
Distance to nearest high-level facility (km)					0.97 (0.92 - 1.02)	0.237
Antenatal care						
4 - 7 visits					0.71 (0.48 - 1.06)	0.093
8 or more visits					0.56 (0.21 - 1.51)	0.253
Multiple gestation					4.69 (2.56 - 8.60)	< 0.001
Previous caesarean					0.17 (0.04 - 0.75)	0.020
Previous miscarriage					0.92	0.784

	(0.49 - 1.72)	
Gestational complications	1.63<br>(0.94 - 2.82)	0.081
Gave birth in-transit	4.02<br>(0.49 - 33.16)	0.197

Appendix 21 legend: Column (1) presents results from unadjusted model, column (2) presents results from model adjusted for demographic characteristics, and column (3) presents results from model adjusted for demographic and obstetric characteristics. All models estimated with standard errors adjusted for clustering at the enrollment antenatal facility. Gestational complications include diagnosis of anemia, hypertension, and/or diabetes during pregnancy. Travel quartile ranges: Quartile 1 = [2.4 km – 16.4 km]; Quartile 2 = [16.4 km – 25.3 km]; Quartile 3 = [25.3 km – 42.3 km]; Quartile 4 = [42.3 km – 435.7 km]. Abbreviations: km = kilometers; UMFL = upward movement in facility level

Appendix 22. Proportion of those who experienced UMFL who ended their delivery episode at a Level 4 facility by total travel distance quartile.

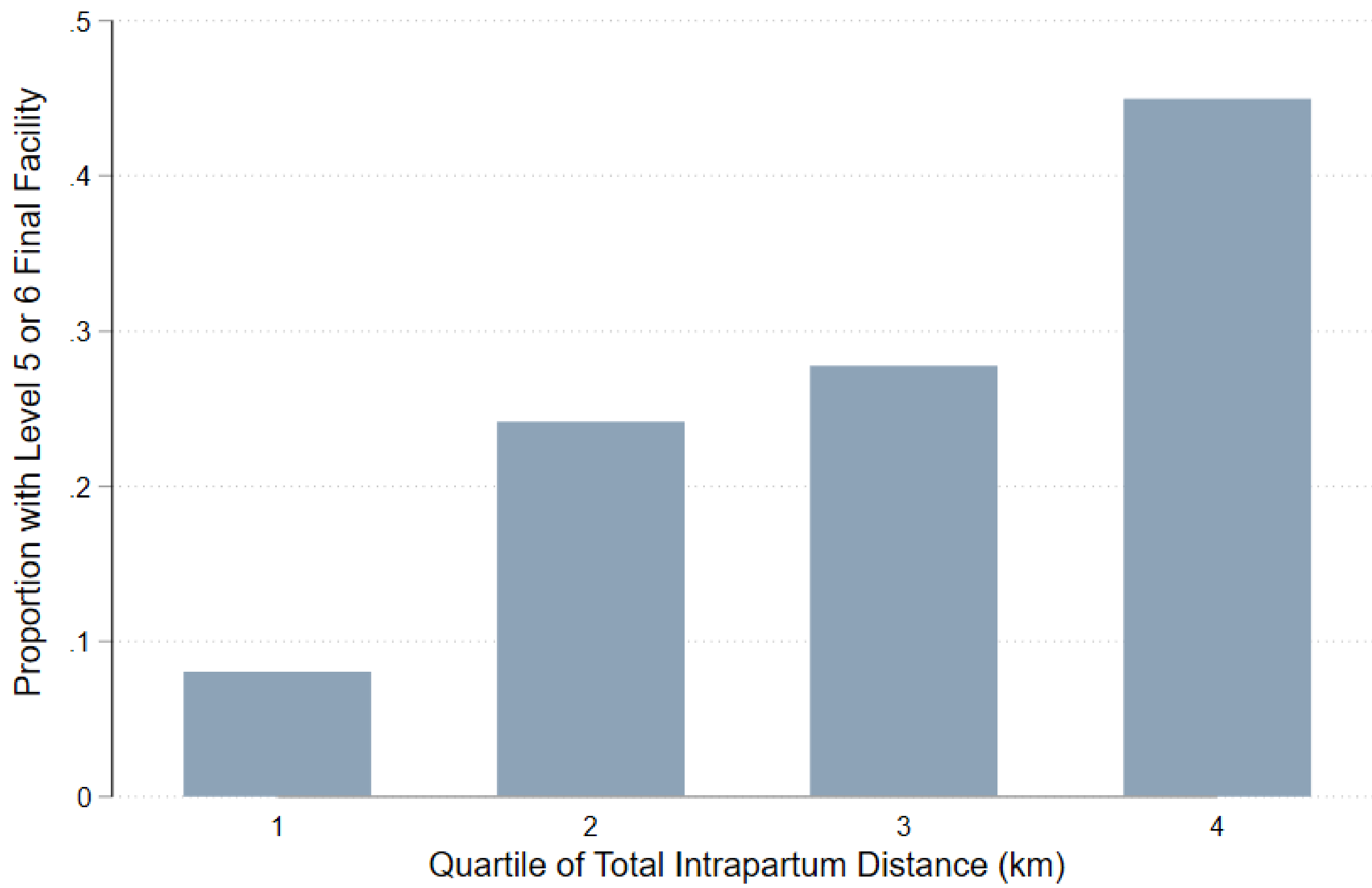

Appendix 21 legend: Quartile 1 = [2.4 km – 16.4 km]; Quartile 2 = [16.4 km – 25.3 km]; Quartile 3 = [25.3 km – 42.3 km]; Quartile 4 = [42.3 km – 435.7 km]. Abbreviations: UMFL = upward movement in facility level.

Appendix 23. STROBE checklist

	Item No	Recommendation	Page No
**Title and abstract**	1	(*a*) Indicate the study's design with a commonly used term in the title or the abstract	
		(*b*) Provide in the abstract an informative and balanced summary of what was done and what was found	
**Introduction**			
Background/rationale	2	Explain the scientific background and rationale for the investigation being reported	
Objectives	3	State specific objectives, including any prespecified hypotheses	
**Methods**			
Study design	4	Present key elements of study design early in the paper	
Setting	5	Describe the setting, locations, and relevant dates, including periods of recruitment, exposure, follow-up, and data collection	
Participants	6	(*a*) *Cohort study*—Give the eligibility criteria, and the sources and methods of selection of participants. Describe methods of follow-up<br>*Case-control study*—Give the eligibility criteria, and the sources and methods of case ascertainment and control selection. Give the rationale for the choice of cases and controls<br>*Cross-sectional study*—Give the eligibility criteria, and the sources and methods of selection of participants	
		(*b*) *Cohort study*—For matched studies, give matching criteria and number of exposed and unexposed<br>*Case-control study*—For matched studies, give matching criteria and the number of controls per case	
Variables	7	Clearly define all outcomes, exposures, predictors, potential confounders, and effect modifiers. Give diagnostic criteria, if applicable	
Data sources/ measurement	8*	For each variable of interest, give sources of data and details of methods of assessment (measurement). Describe comparability of assessment methods if there is more than one group	
Bias	9	Describe any efforts to address potential sources of bias	
Study size	10	Explain how the study size was arrived at	
Quantitative variables	11	Explain how quantitative variables were handled in the analyses. If applicable, describe which groupings were chosen and why	

**Results**			
Participants	13*	(a) Report numbers of individuals at each stage of study—eg numbers potentially eligible, examined for eligibility, confirmed eligible, included in the study, completing follow-up, and analysed	
		(b) Give reasons for non-participation at each stage	
		(c) Consider use of a flow diagram	
Descriptive data	14*	(a) Give characteristics of study participants (eg demographic, clinical, social) and information on exposures and potential confounders	
		(b) Indicate number of participants with missing data for each variable of interest	
		(c) *Cohort study*—Summarise follow-up time (eg, average and total amount)	
Outcome data	15*	*Cohort study*—Report numbers of outcome events or summary measures over time	
		Case-control study—Report numbers in each exposure category, or summary measures of exposure	
		Cross-sectional study—Report numbers of outcome events or summary measures	
Main results	16	(*a*) Give unadjusted estimates and, if applicable, confounder-adjusted estimates and their precision (eg, 95% confidence interval). Make clear which confounders were adjusted for and why they were included	
		(*b*) Report category boundaries when continuous variables were categorized	
		(*c*) If relevant, consider translating estimates of relative risk into absolute risk for a meaningful time period	
Other analyses	17	Report other analyses done—eg analyses of subgroups and interactions, and sensitivity analyses	
**Discussion**			
Key results	18	Summarise key results with reference to study objectives	
Limitations	19	Discuss limitations of the study, taking into account sources of potential bias or imprecision. Discuss both direction and magnitude of any potential bias	
Interpretation	20	Give a cautious overall interpretation of results considering objectives, limitations, multiplicity of analyses, results from similar studies, and other relevant evidence	
Generalisability	21	Discuss the generalisability (external validity) of the study results	
**Other information**			
Funding	22	Give the source of funding and the role of the funders for the present study and, if applicable, for the original study on which the present article is based	
Statistical methods	12	(*a*) Describe all statistical methods, including those used to control for confounding	
		(*b*) Describe any methods used to examine subgroups and interactions	

		(*c*) Explain how missing data were addressed	
		(*d*) *Cohort study*—If applicable, explain how loss to follow-up was addressed<br>*Case-control study*—If applicable, explain how matching of cases and controls was addressed<br>*Cross-sectional study*—If applicable, describe analytical methods taking account of sampling strategy	
		(*e*) Describe any sensitivity analyses	

*Give information separately for cases and controls in case-control studies and, if applicable, for exposed and unexposed groups in cohort and cross-sectional studies.
